# Lipase catalysis in organic solvents: advantages and applications

**DOI:** 10.1186/s12575-016-0033-2

**Published:** 2016-01-13

**Authors:** Ashok Kumar, Kartik Dhar, Shamsher Singh Kanwar, Pankaj Kumar Arora

**Affiliations:** Department of Biotechnology, Himachal Pradesh University, Shimla, 171 005 India; Departmentof Microbiology, University of Chittagong, Chittagong, Bangladesh; School of Biotechnology, Yeungnam University, Gyeongsan, 712-749 Republic of Korea

**Keywords:** Lipase, Organic media, Biocatalysis, Transformations, Industrial applications

## Abstract

Lipases are industrial biocatalysts, which are involved in several novel reactions, occurring in aqueous medium as well as non-aqueous medium. Furthermore, they are well-known for their remarkable ability to carry out a wide variety of chemo-, regio- and enantio-selective transformations. Lipases have been gained attention worldwide by organic chemists due to their general ease of handling, broad substrate tolerance, high stability towards temperatures and solvents and convenient commercial availability. Most of the synthetic reactions on industrial scale are carried out in organic solvents because of the easy solubility of non-polar compounds. The effect of organic system on their stability and activity may determine the biocatalysis pace. Because of worldwide use of lipases, there is a need to understand the mechanisms behind the lipase-catalyzed reactions in organic solvents. The unique interfacial activation of lipases has always fascinated enzymologists and recently, biophysicists and crystallographers have made progress in understanding the structure-function relationships of these enzymes. The present review describes the advantages of lipase-catalyzed reactions in organic solvents and various effects of organic solvents on their activity.

## Background

Enzymatic transformation in organic solvents is an emerging area of research for production of various industrial products. From the processing and economic point of view, the high boiling point and low vapor pressure of water result in expensive purification from an aqueous based biotransformation system. Also, unwanted side reactions such as hydrolysis, racemization, polymerization and decomposition often occur in water which limits many of the reactions of interest in enzymatic synthesis. Organic solvents are extremely harsh to living cells because they are able to bind to the cell membrane and affect its integrity as well as stability. These can disrupt the membrane and decrease the permeability of the barrier which leads to cellular metabolism damages, growth inhibition and finally to the cell death [1–3]. Despite all these worse effects organic solvent-tolerant bacteria are capable of thriving in the presence of these toxic solvents [4].

Researchers used many methods to modify enzymes in order to make suitable them to organic solvents, but instead of these expensive modifications, it would be more desirable to screen the microbes directly for solvent-tolerant [5]. There are several reports on solvent-tolerant lipases classified by the degree of solvent tolerance, which were exploited for industrial use [3, 5–21]. An organic solvent-tolerant lipase from olive oil-induced *Aspergillus niger* MYA 135 was purified using series of chromatography methods [22]. These enzymes are not only stable in these toxic solvents but also capable of catalyzing many synthetic reactions and considered to be most favorable tools for synthetic reactions in non-aqueous systems [23–26]. The microorganisms belonging to *Bacillus*, *Rhodococcus*, *Staphylococcus* and *Arthrobacter* species are tolerant to very toxic organic solvents [16, 27–29]. The enzymes, which can work optimally in harsh conditions such as wide pH range, high temperature, varying salt conditions and in the presence of toxic organic solvents without losing the activities are needed to be explored. The thermophilic enzymes in comparison to mesophilic enzymes display higher resistance to unfavorable conditions and withstand in extreme environments [30]. A particular interest relies on the capacity of a lipase to catalyze such reactions and consequently making possible the synthesis of fine compounds used for manufacturing products of high cumulative value, e.g. biotransformation of oils and fats [31–34]. The lipases can be selected based for a particular transformation based on its activity, stability and selectivity [35, 36]. Many of the industrial processes as well as solubility of substrates/products decide the reaction rate and increase the product yield in biosynthetic reactions [37]. Furthermore, lipases that can function as biocatalysts in nearly anhydrous organic solvents offer new possibilities such as shifting of the thermodynamic equilibrium in favor of synthesis, enable the use of hydrophobic substrates, control or modify enzyme selectivity by solvent engineering, suppress undesirable water dependent side reactions, improve thermal stability of the enzyme(s) and also minimize the chances of contamination.

These advantages are often limited by the low stability and/ or activity of biocatalysts in organic systems. Since most lipases easily get denatured in organic solvents and therefore lose their catalytic activities, thus it becomes pertinent to find lipases that are stable in non-aqueous systems [38, 39]. In order to test solvent toxicity on the microbial cells and organelles, log P, a parameter for solvent hydrophobicity was established which is helpful to determine the stability of proteins and enzymes [40]. The solvents with log P less than 5 are considered harmful to cell membrane because high degree of partitioning can damage the lipid membrane bilayer [4].

## Lipase behavior in organic solvents

Lipase behavior in organic solvents is related with their capacity in both synthetic and hydrolytic reactions. It has been found that *Bacillus* spp. lipases are very stable in hydrophobic organic solvents and their activity is slightly increased in the presence of 10–50 % (v/v) of short chain alkanes, benzene and toluene [41, 42]. It has also been found that the different lipases behave differently in different organic solvents with different level of resistance in different reaction systems [43]. It is worth noting that not only the log P alone but cumulative effect of various other parameters such as the dielectric constant, dipole moment, hydrogen binding and polarizability which affect the enzyme activity in organic solvent system [44]. Beside log P value the solvent polarity, denaturation capacity [44], hydrophobicity [45] and polarity index [32] are also the major factors which decide the stability and catalytic potential of a biocatalyst in an organic medium. However, so far none of them have enabled to validate any serious predictive analysis about catalysis in organic solvents [46].

Lipases differ in their sensitivity towards different organic solvents and are generally more unstable in polar water miscible-solvents than in water-immiscible solvents [47]. Therefore, efforts are being made to screen the enzymes which can work efficiently with high activity in different types of toxic organic solvents. Some bacterial strains are capable to survive in the presence of short chain alcohols like butanol and also in other toxic solvents like benzene and toluene. Two organic solvent tolerant bacteria *Enterococcus faecalis* and *Clostridium sporogenes* were screened in a medium supplemented with acetone or butanol (5 %; v/v) and *S. haemolyticus* was screened in a medium overlaid with 15 % benzene and 85 % cyclohexane. *S. haemolyticus* was able to grow in the presence of cyclohexane, benzene and toluene [29].

## Solvent-tolerant bacteria and lipases

An extremophile, *Bacillus lichiniformis* a solvent-tolerant bacterium which could grow at 55oC was isolated in the presence of different organic solvents [28]. A benzene-tolerant *Rhodococcus* spp. isolated from a contaminated site in Australia on benzene [27], *Rhodococcus opacus* B-4, B-9 and B-10 strains were found to be tolerant to high benzene concentrations *R. opacus* B-4 was highly tolerant to a variety of organic solvents including n-alkanes, alcohol, mono-aromatics and was able to survive with remarkable metabolic activity in neat organic solvents. Lipase purified from *Brevibacillus agri* 52 was found to be stable in various solvents such as up to 90 % diethylenglycol, glycerol and 1,2-propanediol at 37 °C for at least 1 h and the stability decreased to 20 % in 12 h. This lipase showed a poor stability in dimethylsulfoxide (DMSO; 40 %), and displayed 80 % loss in activity after 1 h. An arsenic resistant *Bacillus* sp. ORAs2 which was able to grow in the presence of toluene and benzene was isolated from polluted sediments [48]. A toluene-tolerant *Bacillus cereus* R1 was isolated in an atmosphere saturated with toluene [49]. Gram-positive organic solvent-tolerant bacteria were also isolated by incubation of a suitable agar medium with benzene for several days. The major bacterial strains isolated by this method were *Arthrobacter* ST-1, *Bacillus* sp. DS-994, and *Bacillus* sp. DS-1906 [50–52]. Recently, a new strain of *Staphylococcus xylosus* was isolated using tributyrin or olive oil emulsion and lipase purified from the same strain contained the activity 6300 U/mg at pH 8.5 and 55oC. It showed the highest overall identity (98.74 %) with S. xylosus lipase (SXL1) by N-terminal sequencing [53]. Some strains of Gram positive bacteria were adapted to solvent rich environment sequentially by increasing the concentration of solvents in the culture medium [54]. An attempt to get solvent resistant strain by sequentially transferring the culture into medium containing increasing concentrations of ethanol. This stepwise adaptation eventually gave rise to cells that tolerated up to 8 % (w/v) ethanol and also the enzyme produced by the same culture. Some lipases were noticed to be highly stable in toxic organic solvents even at very high concentration. The purified lipase of *P. aeruginosa* LST-03 [55] exhibited high activity in n-decane, n-octane, (DMSO), N, N-dimethylformamide (DMF) and weak hydrolysis in chloroform, 1-hexanol, 1-pentanol, 1-heptanol, 1-decanol, 1-octanol, 1-butanol, and benzene. The reduced activity of lipase in p-xylene, methanol, toluene or ethanol was not due to denaturation because the half-life of the enzyme in these solvents was more than 7 days. Therefore, these organic solvents specifically decreased the hydrolytic activity of the LST-03 lipase. In the last decade some other organic solvent-tolerant lipases and esterases have been isolated from various microorganisms such as *Pseudomonas* spp., *Burkholderia cepacia* strains, *Bacillus* spp., thermophilic archaea, fungi and yeast [56]. For example *P. aeruginosa* LST-03, B. cepacia ST-200 [25], *Bacillus sphaericus* 205y [57], *Staphylococcus saprophyticus* M36 [24] and *Geobacillus thermoleovorans* CCR11 [58] were also reported to be organic solvent-tolerant bacteria.

## Advantages of biocatalysis in organic solvents

**Fig. 1:**
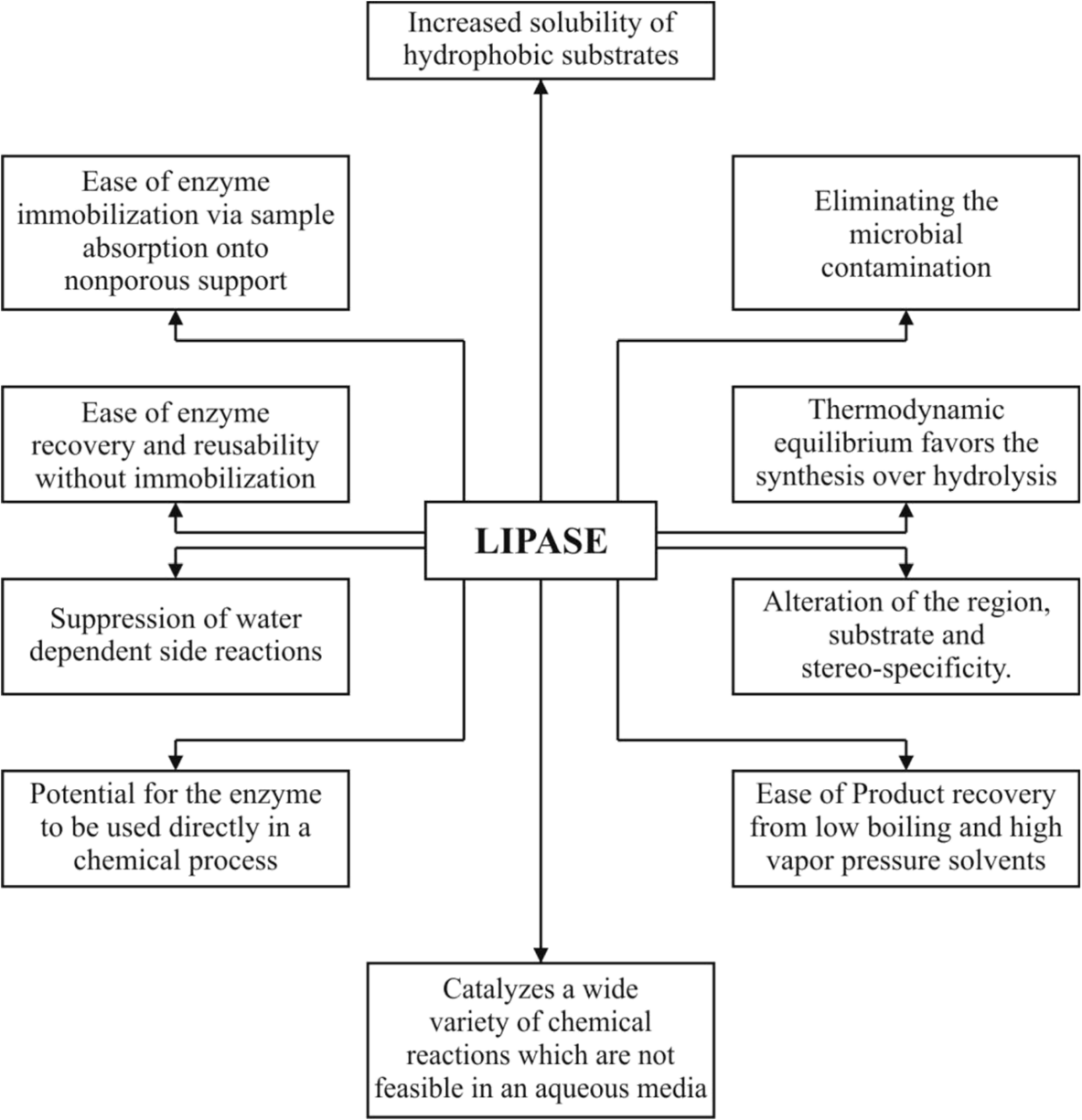
Various advantages of lipase catalyzed reactions in organic solvents. These include a wide variety of chemical reactions which are not feasible in aqueous solutions and also several other applications

Many enzymes are easily denatured and inactivated in the presence of organic solvents. Therefore, protein engineering and several physical and chemical methods, such as immobilization, modification and entrapment for stabilizing enzymes in the presence of organic solvents have been developed [58–60]. Literature survey revealed that extracellular enzymes secreted by organic solvent-tolerant microorganisms for their growth are stable in the presence of organic solvents. On the basis of this hypothesis, researchers screened organic solvent-tolerant microorganisms which produced lipolytic enzymes and succeeded in isolating an organic solvent tolerant *Pseudomonas aeruginosa* LST-03, which secreted solvent-tolerant lipolytic enzyme. Enzymatic reactions in organic solvents provide numerous industrially attractive advantages, such as increased solubility of non-polar substrates, reversal of the thermodynamic equilibrium of hydrolysis reactions, suppression of water-dependent side reactions, alternation of substrate specificity, enantio-selectivity and elimination of microbial contamination. However, the application of enzymes in organic media is still restricted because most enzymes are less active and stable in the presence of organic solvents. Non-polar solvents probably shift the equilibrium from closed to open conformation of the enzyme and also modify the solubility of the substrates and products in the reaction medium. Polar water-miscible solvents are more destabilizing to proteins than water-immiscible solvents, as they remove the salvation water from the enzyme [61]. Good stability in polar solvents is also related with substrate inhibition in the synthesis of flavor esters where one of the reactants is usually methanol or ethanol. Besides bacterial lipases Penicillium expansum lipase has received attention from researchers in catalyzing biodiesel production from corn oil [62] and waste oil [63] in organic media. In a previous study the catalytic activity of this enzyme in both ionic liquid and organic solvent systems have been explored used in catalyzing the methanolysis of corn oil in the ionic liquids [7]. There are many advantages for performing biocatalysis in organic solvents (Fig. 1).

## Factors contributing to the loss of enzymatic activity in organic media

**Fig. 2:**
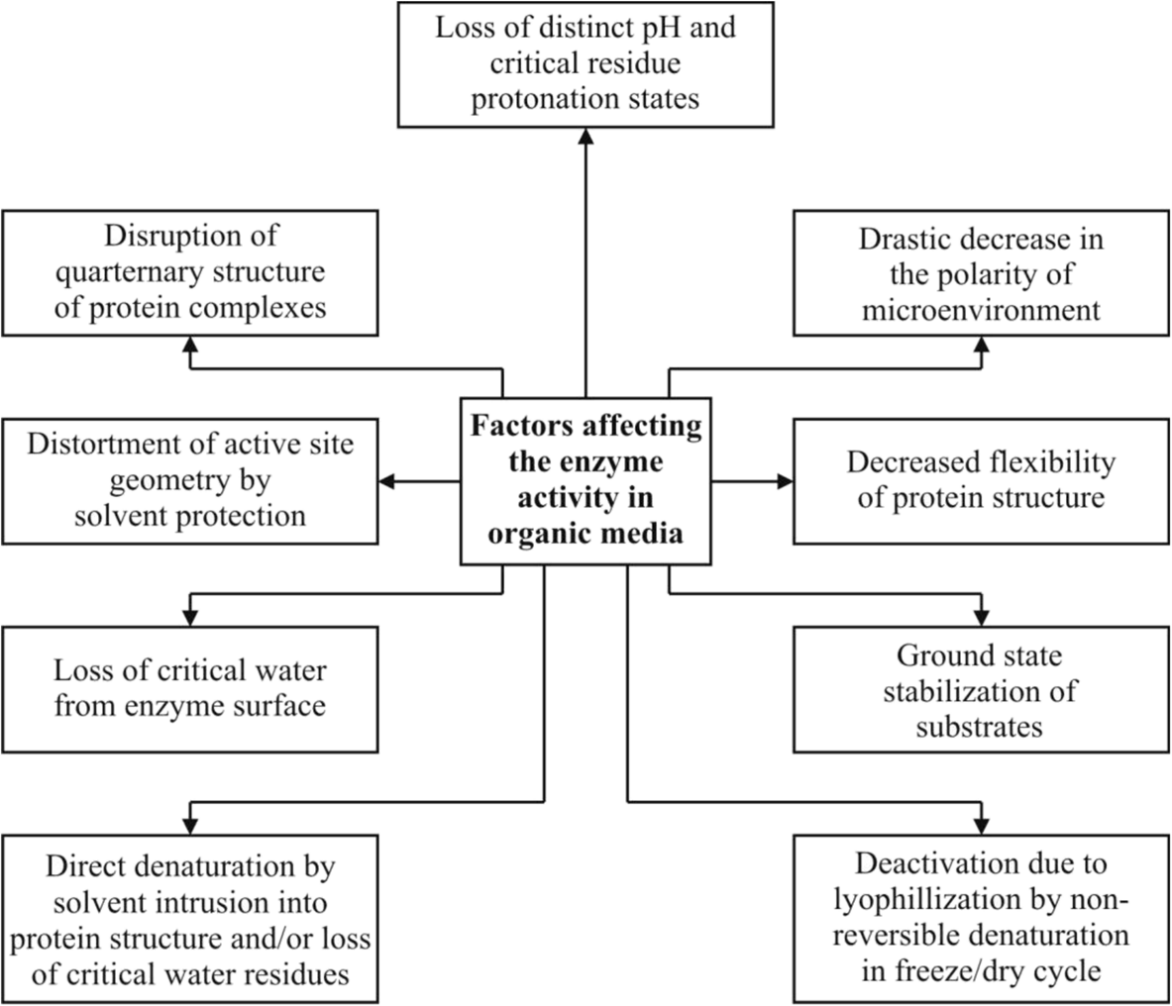
Factors contributing to the deactivation and decrease in the activity of a lipase in organic media. These factors include changes on structure and physiology of lipase

Many factors appear to adversely affect the catalytic function of enzyme in organic solvents. Nonetheless, the situation is not hopeless, as vast majority of techniques have been developed to date to overcome the poor enzyme activity in non-aqueous media. As far as the sensitivity of lipase and lipase producing microorganisms towards different solvents is concerned, lipases and esterases are diverse in their sensitivity to solvents, there is a tendency for water-miscible solvents to cause more significant enzyme inactivation than water-immiscible solvents. In contrast, the enzymes from *Pseudomonas* sp. S5 [64], *B. Sphaericus* 205y and *Arthrobacter nitroguajacolicus* Ru61 [65] were inactivated by the addition of a highly hydrophobic solvent such as hexadecane. This might be due to the relatively high viscosity of the solvents, which hindered efficient interaction between the enzymes and substrates [57]. On the other hand, significant enzyme activation by the addition of organic solvents was observed in the cases of lipases from *P. aeruginosa* LST-03, *Pseudomonas* sp. S5, *B. sphaericus* 205y and *Bacillus megaterium* [66]. The activation of lipase in the presence of some water-miscible organic solvents, such as 2-propanol, can be explained possibly by the disruption of aggregates formed between the enzyme and lipids of the fermentation medium or between the enzyme molecules themselves [67]. A lot of work has been performed to gain a deeper insight into adverse effect of organic solvents in enzyme inactivation which may be attributed to a variety of factors (Fig. 2).

## Thermal stability and solvent tolerance of lipases

High thermal stability of the enzymes is considered to be positively correlated with the stability of enzyme in organic solvents. Thermophilic as well as hyperthermophilic microorganisms are host for many useful enzymes. Archaea is the major source for these enzymes. Thermostable lipases have tendency to get inactivated easily in the water miscible solvents than in the water immiscible solvents. First organic solvent-tolerant lipase was purified from *Pseudomonas aeruginosa* (LST-03) and it exhibited high activity in n-dacane, n-octane and DMSO. In continuation with the research of solvent tolerant lipase after the finding of *P. aeruginosa* (LST-03), other bacteria such as *Bacillus* spp., and *Pseudomonas* spp. were also screened for the production of lipase in the presence of highly viscous solvents. It has been found that *Pseudomonas* sp. S5, *B. spharericus* 205Y and *A. nitroguajacolicus* Ru61a were inactivated by the addition of a highly hydrophobic solvent hexadecane because high viscosity hindered the interaction between substrate and enzyme.

## Various mechanism of inactivation of lipase in organic solvents

It has been found that the disulfide bond and amino acid residue(s) located on the surface of the molecule plays an important role in the organic solvent stability and it is closely related to secondary structure of enzyme molecule. It is generally known that lipase in the hydrophobic solvent gives higher activity than in the hydrophilic solvents. Therefore, conformational mobility of the proteins at such low water content is generally restricted. It means that proteins are more rigid in the non-aqueous system than in the water. When lipases are subjected to act in an organic medium along with conformational changes several factors are responsible for decrease or complete loss in their catalytic activity.

## Changes in cell morphology

In Gram negative bacteria the presence of toxic organic solvents causes an increase in the saturation of membrane fatty acids that enhanced the rigidity of cell membrane which is known as ‘homeoviscous adaptation’ [68, 69]. While in Gram-positive bacteria like, *S. haemolyticus*, the membrane fluidity changes by the change in fatty acid composition which were cultivated in the presence of toluene, benzene or cyclohexane [29, 70]. The chain length of alcohol and other solvent(s) also affects the degree of saturation of membrane fatty acids. In *E. coli* and *P. putida* it has been observed that the presence of long-chain alcohols increases the degree of saturation [71], meanwhile the presence of short-chain alcohols, correspondingly decreases the degree of saturation which leads to an alteration in enzyme production and activity [69, 71]. Bacteria tend to adapt themselves to the presence of toxic organic solvents by perturbation on cell morphology which implicated the major changes in fatty acid and protein composition of cell membrane and also altered the metabolic activities [29, 72]. The changes in membrane protein composition have also been reported in response to solvent stress in Gram positive bacteria. A strain of *Clostridium thermocellum* 27405 adapted to ethanol and its wild-type have been compared for their membrane protein composition. It was observed that 60 % of the proteins identified from *C. thermocellum* 27405 purified membrane fractions were observed to be differentially expressed. In wild strain, as well as in ethanol-adapted strain, the membrane associated proteins were either synthesized in lower amount or were not incorporated properly into the cell membrane [54]. There is no single type mechanism that could confer adaptation to organic solvents but possibly a complex group of mechanism makes the bacterial cell to survive [2]. Thus a combination of different metabolic strategies leads to cellular solvent-tolerance. At genetic level it has been proposed that some bacteria regulate the induction of general stress regulon which induces the production of organic solvent emulsifying or deactivating enzymes [1, 73]. In organic solvent-tolerant bacteria the changes in the lipids and polysaccharides contents of outer membrane after exposure to organic solvent [74, 75] and filamentous growth have also been observed to play a significant role in response to resistant against various solvents [76].

## Regulation by efflux pumps against organic solvents

In some Gram-negative bacteria, the presence of periplasmic space between inner and an outer membrane helps to establish efflux pumps in response to organic solvents [77]. A complex group of efflux pumps plays a major role in the solvent tolerance of bacteria in the toxic environment. These efflux pump systems regulate the concentration of ions in the intracellular environment as well as outer region of the cell in excess of solvent, and thus favour the survival of microorganisms [73, 78]. The adaptation of microorganism is not a result of sudden exposure to solvent rich environment however, during course of evolution, as the bacteria have been exposed to different toxic solvents, the microbes in order to survive and protect themselves evolve a range of metabolic mechanisms to detoxify and minimize the lethal effect of these solvents. Efflux pumps work to extrude out the structurally and functionally unrelated compounds from the bacterial cytoplasm to the external medium [79, 80]. The physical characteristics like charge, hydrophibicity and van der Waals interactions of the solvents and compounds with active sites of enzyme in target proteins determine the regulation of efflux systems [81]. A number of strains of *P. putida* have been found to be tolerant to highly toxic solvents such as p-xylene (log P 3.15), styrene (log P 3.0), octanol (log P 2.92) and toluene (log P 2.69) [75, 82–84]. In *P. putida* srpABC (solvent-resistant pump), ttgABC and ttgB (toluene tolerance genes) encode for the proteins which were similar to AcrAB-TolC efflux pump of E. coli and the MexAB-OprM multidrug efflux system of *P. aeruginosa* [82, 85]. Thus all these solvent resistant systems developed as a result of evolutionary changes and adaptations undertaken by the microbes to live under extreme conditions.

## Conformational changes in the tertiary structure of enzyme

The three dimensional structure of an enzyme is maintained by an intricate balance between hydrophobic interactions, electrostatic charge interactions, hydrogen bonding, disulfide linkages and van der walls forces. In the presence of organic solvents, especially the polar solvents which can penetrate into the active site of enzyme the unfolding of proteins occur due to disturbances in these forces. Conformational flexibility of proteins is also regarded as a crucial determinant of protein function [86, 87]. Enzymes require some essential water bound to the surface of enzymes to exhibit both conformational flexibility and enzymatic activity. Therefore, enzymes are less active in anhydrous solvents than in water due to the restricted conformational flexibility (rigidification of enzyme conformations). Adding water to the enzyme suspension in anhydrous solvents can dramatically enhance enzymatic activity.

## Deformation of active site geometry by organic solvents

As described above, some water is crucial for maintaining protein structure and function. In general, hydrophobic solvents possess less ability to strip the essential water off enzyme molecules than hydrophilic solvents. Therefore, hydrophobic solvents are usually superior to hydrophilic ones for promoting enzymatic reaction in anhydrous solvents. A layer of water or water shell bound to the protein by hydrogen bonds is important for protein function and for retaining the three dimensional structure as well as activity as it represents an integral part of protein structure [88]. But in the presence of organic solvents this hydration shell or layer of water is displaced by organic solvent that results in a dramatic change of protein structure on deformation of active site geometry resulting in loss of activity. The effect of organic solvents on lipase catalysis has been studied by various researchers and it was observed that the rates of reaction are greater in hydrophobic solvents than in hydrophilic solvents [89]. It has been reported that hydrophobic solvents like 1,4-dioxan, THF and higher alcohols are strong denaturants causing inactivation at concentrations as low as 10–30 % volume as compared to hydrophilic solvents like glycerol, ethylene glycol which can be used at concentrations of 50–60 % volume [88]. But in hydrophobic solvents the lipases have been reported to retain higher activities. Because solvent affects the thermodynamic activity coefficient of the substrate or the ground-state free energy of the substrate. Even if the physical nature like viscosity and density of the solvent does not affect the enzyme-substrate interaction, the activation energy barrier is bound to be different in various solvents. It becomes low due to the differences in the ground-state free energy, thus ultimately it makes the lipase catalysis or biotransformation reaction to happen in organic media [90]. It has been argued that the lower activities in organic solvents are due to the restricted flexibility of protein in these solvents [91]. But ultimately if the presence of sulphur bonds and other electrostatic interaction allows the amino acids at active site to retain its original functional conformation then the enzyme can be used in an organic system easily.

## Thermodynamic stabilization of ground state of the substrate

The energy involved in the substrate binding to the enzyme is a major driving force for the enzymatic activity. For substrate binding to occur, there must be desolvation in the enzyme active site. Many enzymes have hydrophobic active sites which have an energetic incentive for hydrophobic substrates to partition from water into the active site. When water is replaced with an organic solvent, the ground state of a hydrophobic substrate is thermodynamically stabilized in organic solvents relative to water. The altered thermodynamics of the reaction medium results in a decrease of the observed enzymatic activity.

## Interfacial inactivation

In an aqueous-organic solvent two phase system, the interaction of enzyme with the bulk organic medium results in inactivation of the enzyme [92]. During biocatalysis in interfacial system, an unfavorable effect can be caused because enzyme molecules adsorb at the interphase, which leads to enzyme denaturation and inactivation as a result of interfacial tension. These interfaces destabilize the electrostatic, hydrophobic and hydrogen bonds interactions of the protein, leading to enzyme irreversible denaturation [93].

## Correlation between enzyme activity and the nature of organic solvents

There have been many attempts to rationalize the correlation between enzyme activity and the nature of organic solvents. Conventional wisdom dictates that water is required for enzyme action. This conclusion originates from the fact that water participates (directly or indirectly) in all non-covalent interactions maintaining the native, catalytically active enzyme conformation [94, 95]. Hence, the complete removal of water should drastically distort that conformation and inactivate the enzyme. Although this reasoning is undoubtedly correct, the real question is whether water is indeed required but how much water is crucial to retain the catalytic activity of lipase. As long as this water is present around the enzyme molecules, the rest of water can probably be replaced with an organic solvent without adversely affecting the performance of the enzyme. Since the absolute amount of water present in a few monolayers is very small, this situation is as good as to an enzyme functioning in a nearly anhydrous organic medium. Stability of lipases in organic solvents makes their uses commercially feasible in the enzymatic esterification reactions [96–99].

As far as the conformational properties and functioning of lipases in organic solvents is concerned, lipases were found to be fit because of their rigid conformation and interfacial activation characteristics. Many lipases are active in organic solvents where they catalyze a number of useful reactions including esterification [100–102]. Various factors for maintaining lipase activity in non-aqueous media have been considered such as tuning up of the lipases by pH and it is accomplished while the enzyme is dissolved in the buffer prior to dehydration (lyophillization) before its suspension in organic solvent. Also the correct protonation state of the side chain of amino acids residues of lipase is important for retaining its catalytic activity [103]. A relevant practical example is the use of esterses and lipases to catalyze esterifications in organic solvents such as isopropyl acetate, ethyl ferulate, isopropyl ferulate and butyl ferulate [104–106]. Enzymatic reactions in organic media are actually divided into two systems: reactions performed in organic solvent systems and in solvent-free systems. The solvent-free system, i.e. the reaction mixture comprising only liquid organic substrates (such as liquid oil) without any organic solvent, if it is possible, has high volumetric performance and economic advantages over the organic solvent system especially for large scale production. It is also desirable for the synthesis of food-grade products since very stringent safety regulations concerning organic solvent usage have to be observed in food industry. Non aqueous enzymology is concerned with the utilization and understanding of enzymes in essentially organic environments. Conventional biocatalysis is carried out in aqueous media, and it is not surprising that most of the methods developed to study enzymes performance are water based.

But day by day the interest has been focused on using enzymes to catalyze reactions in organic media [107]. If an enzyme could function in an essentially organic environment, increased ease of product recovery, increased hydrophobic reactant solubility and reduced microbial contamination would be properties contributing to its wide applicability. Altering the solvent can have a dramatic effect on enzyme function. Also lyophilized enzyme powders suspended in organic solvents could catalyze a variety of novel catalytic functions e.g. esterification, transesterification, interesterification etc. The exclusion of water leads to an improved enzyme thermostability (as water is a reactant in many processes that irreversibly denature enzymes) and the reduction of undesirable side reactions that require water as a substrate [108]. There has been much interest in the development of rules to predict the effects of various solvents on the biocatalyst [109]. A good correlation was found between the ester mole fraction at equilibrium and log P of the solvent. The equilibrium constant for esterification correlates well with solubility of water in the organic solvents. The catalyst activity, measured as the initial rate of the esterification reaction, is best correlated as a function of n-octanol-water partition (log P) coefficient; electron pair acceptance index or the polarizability [40]. When log *P* <2, distortion of water structure occurs; if 2< log *P* <4, the effect of solvent is unpredictable and if log *P* >4, water structure is intact. Although the equilibrium position for lipase-catalyzed esterification reactions is independent of the enzyme, it is interesting to note that it is not independent of solvent [110]. The hydrophobic solvents yielded higher reaction rates than the hydrophilic ones even at a constant water activity; however, better enantio-selectivity was observed [111]. Enzymatic methods of ester synthesis are more effective when performed in non-aqueous media [112].

## Advantages of lipase catalysis over chemical reaction(s)

In the times of industrial biotechnology, the leading representatives of the new science promoted the idea of using biological systems to create more efficient, more selective and environment friendly processes for the conversion of raw materials into industrial products, thereby substituting problematic chemical transformations. Therefore, the demand for industrial enzymes particularly of microbial origin, is ever increasing owing to their applications in a wide variety of processes. The catalyst part of the biological system can thereby consists of whole cells, or purified enzyme(s). Enzyme-mediated reactions are attractive alternatives to tedious and expensive chemical methods. In the present scenario, enzymes such as proteases and amylases have dominated the world market owing to their hydrolytic reactions for proteins and carbohydrates. However, with the realization of the biocatalytic potential of microbial lipases in both aqueous and non-aqueous media in the last one and a half decades, industrial fronts have shifted towards utilizing this enzyme for a variety of reactions of immense importance. Major improvements have been achieved in the development and application of lipase as catalyst with the advancement in modern enzymology. Compared with conventional chemical synthesis from alcohols and carboxylic acids using mineral acids as a catalyst, the use of enzymes such as lipases to produce the high value-added fatty acid esters in solvent-free media may offer many significant advantages [105, 106, 113–118].

Environmentally friendly and economic processes are the goals of each and every industry. The use of lipases for the synthesis of chiral drugs, cosmetic or nutritional compounds is well established since many years ago. Lipid modification strategies for industry include processes such as fractionation, hydrogenation and interesterification. While each of the two processes has specific uses and advantages, interesterification reaction offers the greatest potential application. It includes three approaches: acidolysis, alcoholysis and transesterification. Chemically, it can be induced by the use of alkali catalysts in a reaction which lacks specificity and offers little or no control over the positional distribution of fatty acids in the final product [119]. In the chemical synthesis, mineral acids are most commonly used to catalyze the esterification. Other agents such as tin salts, organo-titanates, silica gel and cation-exchange resins are also employed. The classical acid catalysis may lead to unwanted side reactions. Although the metal salts minimize the side reactions, they require higher temperature [120]. Normally, the fat and oil modifications carried out by the chemical inter-esterification are energy intensive and non-specific. The esterification by the lipases appears to be an attractive alternative to the bulk chemical routes. Lipases are being developed to carry out the transformations without the extreme temperature and pressure conditions which are essential for the traditional industrial processes. Lipases display a high degree of specificity and enantio-selectivity for the esterification and transesterification reactions [121] which makes them a principle biocatalyst in the trans- and inter-esterification reactions for the synthesis of several useful acylglycerols. The use of lipase to carry out the esterification alleviates the need for a wide variety of the complex post-reaction separation processes, which may lower the overall operating costs.

## Conclusion

Today, lipases find immense applications in diverse areas of industrial microbiology and biotechnology. This statement is well documented by the enormous number of research investigations undertaken in the last one and a half decades. Lipases show immense versatility regarding their catalytic behavior. Ionic liquids and supercritical fluids provide new opportunities. Lipases themselves may be improved by protein engineering. Rational design of lipases remains largely unexplored, but has the potential for enhancing the enzyme thermo-stability, improving its solvent tolerance, increasing its specificity and heightening its activity. Current commercial use of lipases, together with new applications, will continue to play an important role in maintaining and enhancing the quality of life we enjoy today while protecting the environment for generations to come. However, complete understanding of the different lipases requires greater input of research effort(s). Novel lipases with properties of chemo-, regio- and enantio-selectivity have been isolated, which may be eligible for exploitation at commercial level for industrial applications in course of time. But, some of the indigenously developed technologies for the production of lipases are already in the commercial production stage. Furthermore, comparison of some of the lipases produced by microorganisms indigenously is at par or even better than the well-known commercially available imported lipases. Thus, utilizing these lipases will greatly boost many biotechnology-based industries.
